# Contemporary evolution and the dynamics of invasion in crop–wild hybrids with heritable variation for two weedy life–histories

**DOI:** 10.1111/eva.12366

**Published:** 2016-02-28

**Authors:** Lesley G. Campbell, Zachary Teitel, Maria N. Miriti

**Affiliations:** ^1^ Department of Chemistry & BiologyRyerson UniversityTorontoONCanada; ^2^ Department of Evolution, Ecology and Organismal BiologyThe Ohio State UniversityColumbusOHUSA; ^3^ Present address: Department of Integrative BiologyUniversity of GuelphGuelphONCanada

**Keywords:** agriculture, artificial selection, evolutionary demography, hybridization, invasive species, life table response experiment, life‐history evolution

## Abstract

Gene flow in crop–wild complexes between phenotypically differentiated ancestors may transfer adaptive genetic variation that alters the fecundity and, potentially, the population growth (*λ*) of weeds. We created biotypes with potentially invasive traits, early flowering or long leaves, in wild radish (*Raphanus raphanistrum*) and F_5_ crop–wild hybrid (*R. sativus *×* R. raphanistrum*) backgrounds and compared them to randomly mated populations, to provide the first experimental estimate of long‐term fitness consequences of weedy life‐history variation. Using a life table response experiment design, we modeled *λ* of experimental, field populations in Pellston, MI, and assessed the relative success of alternative weed strategies and the contributions of individual vital rates (germination, survival, seed production) to differences in *λ* among experimental populations. Growth rates (*λ*) were most influenced by seed production, a trait altered by hybridization and selection, compared to other vital rates. More seeds were produced by wild than hybrid populations and by long‐leafed than early‐flowering lineages. Although we did not detect a biotype by selection treatment effect on lambda, lineages also exhibited contrasting germination and survival strategies. Identifying life‐history traits affecting population growth contributes to our understanding of which portions of the crop genome are most likely to introgress into weed populations.

## Introduction

The ecological processes of population growth and persistence are shaped by the evolutionary characteristics of a population, that is, phenotypic frequencies and their relative fitness (Darwin and Wallace 1858; Simpson 1944; Gould 1989). In fact, rapid, adaptive evolution in response to environmental variation is expected to result in altered demography, which has implications for population growth rates. The Galapagos finches (*Geospiza fortis*) experienced catastrophic demographic decline during a drought (Grant and Grant 2002); the population crash was subsequently explained, in large part, by slowed evolution due to genetic load (Hairston et al. 2005). To complement such natural ‘experiments’, reciprocal translocation experiments show that local adaptation can dramatically affect reproductive success (Kinnison et al. 2008; Hereford 2009), a correlation of population growth for many annual plants. Several experimental microcosms have manipulated genetic diversity (presumably neutral and adaptive) to determine that its very presence positively influences population persistence (e.g., in small versus large common toad [*Bufo bufo*] populations, Hitchings and Beebee 1998; predator–prey ecosystems consisting of algae and rotifers, Hitchings and Beebee 1998; Yoshida et al. 2003). Finally, introduction of predators into natural populations of *Poecilia reticulata* resulted in rapid evolution of key phenotypic (e.g., dulled male coloration) and demographic traits (i.e., delayed maturation and fewer, larger offspring (Reznick and Bryga 1987). Thus, trait evolution is likely a significant driver of population demography (Frankham 2005; Kinnison and Nelson 2007). Yet, to our knowledge, there are no published descriptions of experimental manipulations of adaptive trait variation in populations that subsequently explore the consequences for population growth.

Gene flow is one evolutionary mechanism that alters the quality and quantity of adaptive trait variation (Arnold 1997). When gene flow is high, it has a homogenizing effect (Burgess et al. 2005), constrains adaptive evolution (e.g.*,* Slatkin 1987; Kirkpatrick and Barton 1997; Lenormand 2002), and may cause population declines (e.g., Hanski 1999). In contrast, episodic events of gene flow may hasten adaptive evolution (e.g., Ehrlich and Raven 1969; Gomulkiewicz et al. 1999; Rieseberg et al. 2003; Hendry 2004; Whitney et al. 2006) and thus contribute to weedy population growth (Hovick and Whitney 2014). Therefore, gene flow between genetically distinct crops and sexually compatible weedy relatives may contribute to the evolution of more problematic weeds when gene flow alters phenotypic frequencies and the relative fitness of phenotypes within weed populations.

Crop‐to‐wild gene flow has served as a model system to evaluate the ecological and evolutionary consequences of gene flow and the potential for hybridization to lead to rapid evolution of life‐history and fitness‐related traits (e.g., Campbell et al. 2006; Hovick et al. 2012). The impact of crop–wild gene flow depends on rates of gene flow and the relative success of heritable, migrant phenotypes when compared with the recipient population's adaptive optima (Snow et al. 2003; Hooftman et al. 2005; Mercer et al. 2006). Studies on crop–wild hybrid populations show that the successful phenotypes are not always a random subset of genotypes from parental populations (Ellstrand and Schierenbeck 2000; Hovick and Whitney 2014), and hybrid populations may facilitate the transfer of novel, adaptive traits to recipient weed populations (e.g., Snow et al. 2003; Hooftman et al. 2011; Owart et al. 2014). Indeed, over short‐time scales, crop‐to‐wild gene flow can be a more significant source of adaptive genetic variation than mutation (Gomulkiewicz et al. 1999; Holt et al. 2004).

Risk assessment of crop–wild hybridization often explores the evolution of increased fecundity of weeds (Pilson and Prendeville 2004; Snow et al. 2005; Ellstrand et al. 2014). Although it is difficult to predict an invader based on a suite of traits (Perrins et al. 1992), a quintessential, annual weed often reaches sexual maturity quickly or grows large quickly to compete for limited resources (or both, Roff 1992; Stearns 1992). From a demographic perspective, weedy populations may exhibit high population growth rates, a strong capacity to colonize new locations, and/or high population persistence (Campbell et al. 2014). Certainly, a short life cycle will reduce the likelihood of death before reproduction, but individuals may reproduce at a size smaller than is adaptive and therefore curtail reproduction. In contrast, large size at reproduction may not only provide a competitive benefit, but allometric consequences of large size may also result in the production of more offspring (Weiner et al. 2009). Yet, there are few controlled experiments linking genetic variation in life‐history traits to population demographic consequences. Life table response experiments (LTRE) offer a robust tool to measure the demographic significance of life‐history variation in experimental populations (Hooftman et al. 2007; Campbell et al. 2014).

Here, we build on our studies of fitness components (Campbell et al. 2006; Hovick et al. 2012), evolutionary responses of two key life‐history traits to directional selection (Campbell et al. 2009a,b), and demographic analyses of populations experiencing natural selection (Campbell et al. 2014) to determine the influence of heritable variation for early flowering or long leaves (as an indicator of plant size) on the relative population growth of advanced‐generation hybrid and wild radish biotypes grown in a common garden in Michigan, USA.

## Methods

### Study species

We used the crop–wild complex of cultivated radish (*Raphanus sativus*), an open‐pollinated vegetable selected for large, colorful roots and high seed production (Snow and Campbell 2005), and its weedy relative, wild radish (*Raphanus raphanistrum*, also known as jointed charlock), a cosmopolitan, agricultural weed that also colonizes disturbed sites and coastal beaches (Warwick and Francis 2005). These two radish species have emerged as model systems in plant evolutionary ecology and in the assessment of ecological consequences of crop‐to‐wild gene flow (Mazer et al. 1986; Klinger and Ellstrand 1994; Snow et al. 2001, 2010). Although *R*. *raphanistrum* and *R*. *sativus* share many phenotypic characters, they exhibit divergent life histories in several key traits associated with weediness. Many *R. sativus* cultivars germinate quickly and develop large rosettes before bolting and flowering late in the growing season, whereas *R*. *raphanistrum* plants germinate slowly and inconsistently, form narrow, branching taproots and develop smaller rosette sizes before flowering early in the growing season (Panetsos and Baker 1967; Campbell et al. 2009a,b).

Using genotypes from natural selection experiments, we have studied many aspects of crop‐to‐wild gene flow and hybrid fitness of radishes in Michigan, California, and Texas, where *R. raphanistrum* is non‐native and, sometimes, weedy. Unlike in Michigan where wild and hybrid phenotypes were equally successful, hybrids grown in California exhibited ~22% greater survival and ~270% greater fecundity than wild plants. Furthermore, in Texas, hybrids were more successful at colonizing this novel location, due to earlier, increased germination, and increased survival, despite producing fewer seeds per plant (Hovick et al. 2012). Finally, hybrid populations had faster population growth than wild plants in Michigan, under low, but not high, competition conditions (Campbell et al. 2014). These results are consistent with the hypothesis that crop–wild hybrid biotypes have the potential to displace their wild parent in certain environments (Ellstrand and Schierenbeck 2000; Hovick and Whitney 2014).

### Biotypes

Detailed descriptions of the wild and hybrid populations are available in Campbell et al. (2009a,b). Briefly, control and artificially selected populations were generated by hand‐pollinating 100 wild *R. raphanistrum* plants with either wild pollen to create F_1_ wild biotype populations, or pollen from 100 *R. sativus* var. ‘Red Silk’ plants (Harris‐Moran Seed Co., Modesto, CA, USA) to create F_1_ hybrid biotype populations. Based on hybridization in this first generation, we refer to radish biotypes as wild or hybrid. Physical separation and unpollinated control flowers were used to ensure that crosses between these self‐incompatible plants were uncontaminated.

Artificial selection was imposed on glasshouse‐grown plants for three generations (F_2_–F_4_). Randomly mated ‘control’ populations and artificial selection populations were initiated in the F_2_ generation after 100 individuals from each F_1_ biotype were cross‐pollinated (Campbell et al. 2009a,b). During three generations of mating (F_3_–F_5_), populations were initiated with 130–200 F_2_–F_4_ individuals and were propagated with a subset (10%) of individuals from each replicate each generation (Table 1). For the purposes of imposing selection and following trait evolution, we recorded dates of germination and anthesis, and leaf length at anthesis of each plant. Age at flowering was calculated as the difference, in days, between germination and anthesis. As the length of the longest leaf in wild and crop–wild hybrid radish is correlated with several measures of plant size at the time of reproduction (e.g., number of flowers, stem diameter at harvest, Campbell et al. 2009a,b), the length of the longest leaf on the first day of flowering served as an early indicator of plant size at the time of reproduction. Applying truncation selection, we selected 10% of the plants from each lineage that represented the earliest flowering individuals for early lineages, 10% of the plants from each lineage that represented the longest leafed individuals for long lineages, and randomly selected 10% of the plants from the control lineages to produce the following generation.

**Table 1:** Summary of wild and hybrid populations included in this experiment

Biotype	Selection treatment	Number of generations of artificial selection or random mating	Number of populations
Wild	Early‐flowering	3	3
	Control^a^	3	3
	Long‐leaf	3	3
F_5_ Crop–Wild hybrid	Early	3	3
	Control^a^	3	3
	Long‐leaf	3	3

a Note that these populations did not experience selection but rather random mating for three generations.

Selected plants were cross‐pollinated within a lineage in a complete diallel design. To account for drift as a possible evolutionary mechanism, we created three independent populations for each treatment combination (wild or hybrid; early, large or control) for a total of eighteen populations (Table 1). Populations represented the variation in evolutionary trajectories of randomly mated or artificially selected populations typically associated with genetic drift. We assumed that if control populations became adapted to experimental conditions, this had only minor effects on the phenotypic and demographic traits of interest. Thus, we used the control populations to determine the expected variation in traits without selection in advanced‐generation hybrid and nonhybrid populations.

### Demographic experimental design

We measured vital rate dynamics of populations from the wild and hybrid artificial selection populations in a common garden. As in previous studies (Campbell et al. 2009a,b), the common garden was located at the University of Michigan Biological Station in Pellston, Michigan, USA. The proximity of the common garden to our original experimental plots helped to assure that the phenotypic variation observed was typical for these plants (e.g., Campbell et al. 2006). In 2004, we collected F_5_ seeds from F_4_ artificial selection population plants (see Campbell et al. 2009a,b).

The common garden included F_5_ wild and hybrid artificial selection populations. Whole fruits were planted on May 30, 2005 in 3.54 L of local sandy soil in an aluminum foil pan (22.9 cm × 30.34 cm × 5.1 cm, Walmart, Cheboygan, MI, USA) with holes puncturing the bottom surface, allowing plant roots to grow into local soil and excess water to drain easily. The number of seeds within a fruit was estimated based on the number of visible locules from the outside of the fruit. For the artificial selection lineages, we planted six locules per pan. Each artificial selection lineage (e.g., Hybrid Control Rep 1) was represented by five replicate pans (a total of 30 seeds). Pans were arranged in a complete randomized block design. Within a pan, fruits were spaced out as evenly as possible. Pans were separated by at least 30 cm from neighboring pans to minimize root and shoot competition. Pans were watered every other day until August 31st. Insecticide (0.0033% esfenvalerate, 20 g/9.5 L, Scotts Miracle‐Gro Co., Marysville, OH, USA) was used to control insect herbivory three times during the first month after planting, when aphid herbivory was highest. Aphids were present at low densities later in the season but did not colonize any plant heavily. Pollinators were abundant throughout the experiment (as in Lee and Snow 1998). Plants were individually harvested as they senesced, until the first hard frost (September 16th–20th, 2005), when we harvested all remaining plants. Harvested radish plants were dried at 60°C.

### Censuses and data collection

From May 10, 2005 to September 19, 2005, we censused plots weekly to record changes in demographic status of the experimental individuals. Each week, new individuals were flagged to identify them in future censuses and all flagged individuals were categorized as either dead or in one of the three stages mentioned below. Once plants were harvested, we recorded flower number, fruit number, and seeds per fruit as measures of lifetime fecundity. To estimate the number of seeds per plant, we multiplied the average number of locules per fruit (for 10 randomly chosen fruits per plant) by the number of fruits.

### Matrix construction

We classified plants into three stages, chosen after several years of observing this species. The stages were seeds, germinating cotyledonous plants (plants with only cotyledons), and flowering plants (plants with open flowers). Four demographic transitions were included in our model for *Raphanus* populations using data from the 2005 field season: seed dormancy/mortality, germination, survival to flowering, and fecundity. Because our methods could not distinguish between seed dormancy and mortality, we maintain both terms in a single demographic parameter. Lumping these terms was justified by the results of a recent study exploring the seedbank dynamics of *Raphanus raphanistrum* and F_3_ crop–wild hybrids; seed dormancy was ~58% lower in hybrid versus wild populations whereas seed mortality did not differ among biotypes (~8% of seed, Teitel 2014). If the artificial selection lineages used here differed in seed mortality and dormancy, comparing mortality and dormancy between lineages is inappropriate because the effect of seeds in this stage are not equivalent. Note in the results, however, that the relative contribution of mortality and dormancy to lambda is the smallest among all life‐history stages, and this is consistent with our findings in other studies (Campbell et al. 2014; Teitel 2014). Therefore, differences in seedbank dynamics tend to have a relatively small impact on population growth compared with juvenile survival or fecundity. Our analysis synthesizes the dynamic vital rates across the annual summer growing season, from planting on June 7 to harvesting on September 19.

### Matrix algebra, LTRE, and sensitivity analyses

We used a fixed‐effect LTRE (Caswell 2001) to model lambda (*λ*) of each experimental population (18 constructed matrices; e.g., wild early‐flowering replicate 1, hybrid large replicate 2, Appendix 1) as a linear function of biotype (*g*), selection treatment (*s*), and their interaction (*gs*): −*λ* ^*gs*^ = *λ* ^(..)^ + *α* ^*g*^ + *β* ^*s*^ + *αβ* ^*gs*^ where *α* ^*g*^ is the effect of the *gth* level of the biotype, *β* ^*s*^ is the effect of the *sth* level of the selection treatment, and *αβ* ^*gs*^ is the interaction of the *gth* biotype and *sth* selection treatment, measured relative to the projected growth rate of a reference matrix ^(..)^. We obtained our reference matrix by combining data from randomly mating wild or hybrid populations into a mean (calculated by averaging transition frequencies) matrix (Miriti et al. 2001). To obtain the treatment matrices, we first averaged all replicates of matrices belonging to a given treatment combination (e.g., the transition frequencies of wild early replicates 1, 2, and 3 were averaged). We then averaged common treatment groups of these matrices to give us mean representative matrices for a given treatment (mean wild type, mean early flowering). We estimated treatment effects as:

$$\begin{array}{cc} \alpha^{g}= & \;\lambda^{g.}-\lambda^{..}\\ & \;\;\;\;\;\;\;\approx \sum \left[a_{ij}^{g.}-a_{ij}^{..}\right]\cdot \left(\delta \lambda /\delta a_{ij}\right){|}_{[A^{g.}+A^{..}]/2} \end{array}$$

$$\begin{array}{cc} \beta^{s}= & \;\lambda^{.s}-\lambda^{..}\\ & \;\;\;\;\;\;\;\approx \sum \left[a_{ij}^{.s}-a_{ij}^{..}\right]\cdot \left(\delta \lambda /\delta a_{ij}\right){|}_{[A^{.s}+A^{..}]/2} \end{array}$$

$$\begin{array}{cc} \alpha \beta^{gs}=\; & \lambda^{gs}-\lambda^{..}-\alpha^{g}-\beta^{s}\\ & \;\;\;\;\;\;\;\approx \sum \left[a_{ij}^{gs}-a_{ij}^{..}\right]\cdot \left(\delta \lambda /\delta a_{ij}\right){|}_{\frac{\left[A^{gs}+A^{..}\right]}{2}}-\alpha^{g}-\beta^{s} \end{array}$$

where we obtained sensitivities (*δλ/δa*_*ij*_) from the relationship *δλ/δa*_*ij*_ = *v*_*i*_ *w*_*j*_/<***w**,**v***>, and ***v*** and ***w*** are the right and left eigenvectors of the matrix. We then evaluated the sensitivities, halfway between the reference and treatment matrices (Caswell 2001). We obtained treatment matrices (e.g., **A** ^g.^, **A** ^s^) by pooling data across all levels of the other treatments. Finally, the contributions were calculated by weighting the differences in vital rates by their sensitivities. In general, a vital rate will increase or decrease lambda relative to some standard model (i.e., the mean matrix). For instance, a positive contribution of fecundity suggests that fecundity in the ‘experimental’ population made lambda more positive relative to the mean. Therefore, we interpreted the above equations as how both observed variation in matrix elements, and the sensitivity of population growth to variation in those elements, influence the effect of the treatments on population growth. A particular matrix element *a*_*ij*_ may contribute little to variation in lambda in cases when *a*_*ij*_ was invariant among treatment classes or when lambda was insensitive to variation in *a*_*ij*_. Additionally, *a*_*ij*_ may contribute little to variation in lambda even if lambda was highly sensitive to the element if the vital rate did not differ among treatments. In alternate scenarios, even small amounts of variation in *a*_*ij*_ may drive variation in lambda when there are consistent differences among treatments and when lambda is highly sensitive to that matrix element. One must note that contributions can differ in direction and magnitude even if there is no significant difference among lambdas of the experimental and mean matrices (Caswell, 2001). It is important to recognize that these contributions show the consequences of vital rates on population growth and are not a measure of the statistical significance of a vital rate. Matrix algebra and analyses were performed using MATLAB (v.2012a; The Mathworks, Inc., Natick, MA, USA).

### Vital rate comparisons

To test whether the estimates of the three vital rates and lambda were similar among artificial selection wild and hybrid populations, we ran a Type III multivariate anova in which biotype and selection treatment, and their interaction were fixed effects. As the proportion of seeds that remains in the seed bank is correlated with the proportion of seeds that germinate, we only tested the effects of biotype and selection on germination. Germination and survival to flowering were arcsine square root transformed to normalize data; fecundity and lambda were log_10_ transformed. When significant differences were detected, *post hoc* comparisons were performed using Tukey's correction for multiple hypothesis tests. All statistical analyses were performed using SPSS v. 21. (SPSS Inc., Chicago, IL, USA)

## Results

**Figure 1:**
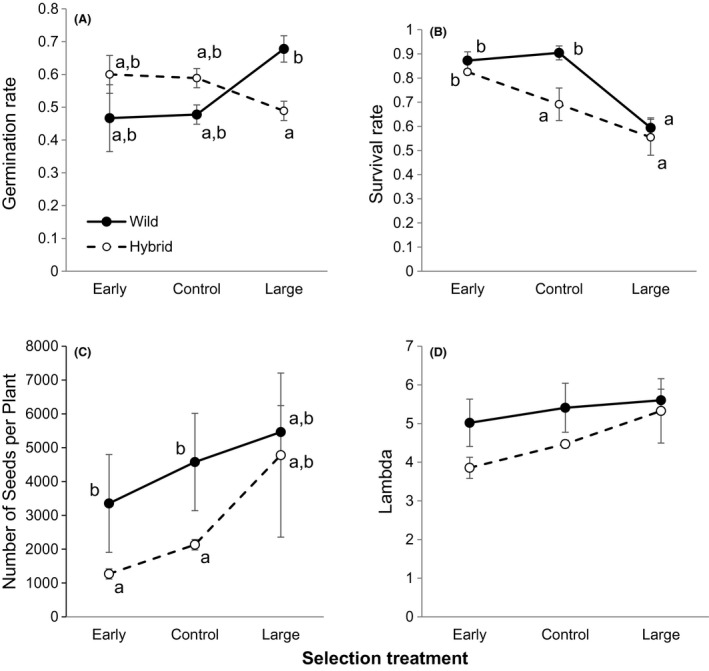
Comparison of least square mean vital rates and population growth rates of wild (solid line) and F_5_ hybrid populations (dashed line) after selection for early flowering or long leaves, relative to random mating grown in a field experiment in Pellston, MI, USA. (A) Germination rate; (B) Proportion of the population that survived to flower; (C) Number of seeds per population; (D) Lambda. Error bars represent the SE of the mean; *n* = 3 replicate populations per biotype and selection treatment combination.

Weed population demography responded to our artificial selection treatments (Multivariate anova,* F*_6,22_ = 2.864, *P* = 0.032), hybrid ancestry (*F*_3,10_ = 2.745, *P* = 0.099), and their interaction (*F*_6,22_ = 2.44, *P* = 0.058). Although all populations exhibited positive population growth, lambda was marginally significantly higher in wild (*λ* mean ± SE = 5.34 ± 0.17) than hybrid populations (4.55 ± 0.43, Fig. 1, Table 2). Whereas, the biotypes did not differ significantly in germination rate or survival to flowering, wild plants produced significantly more seeds than hybrid plants (Fig. 1, Table 2). Population growth rate did not differ between selection treatments, and there were no significant differences in vital rates among selection treatments. Finally, although we did not detect a significant biotype by selection treatment effect on lambda, lineages exhibited contrasting demographic strategies. Germination rates in wild lineages were highest when the population had experienced artificial selection for long leaves whereas the pattern was opposite in hybrid lineages (Fig. 1, Table 2). Survival to flowering was marginally significantly higher for wild control lineages than hybrid control lineages, but did not differ among biotypes across early or long‐leaf treatments. Long‐leafed populations produced marginally more seeds than the early‐flowering lineages (Table 2, Fig. 1). Therefore, demographic growth and relative invasiveness may be significantly altered by genotypic frequencies and artificial selection for weedy traits within populations.

**Table 2:** Summary of *F*‐statistics and *P*‐values (indicated with superscript symbols: *P < 0.05; †*P* < 0.10) from anovas to test for significant differences across biotypes and artificial selection treatments (and their interaction) in the rates of germination, survival to flowering, number of seeds, and population growth (lambda)

Factor	df (numerator, denominator)	Germination	Survival to flowering	Number of seeds	Lambda
Biotype (B)	1, 2	0.03	2.96	27.64*	10.58†
Selection (S)	2, 2	0.10	6.88	19.64*	5.67
B × S	2, 12	5.43*	2.77†	0.17	0.38

**Figure 2:**
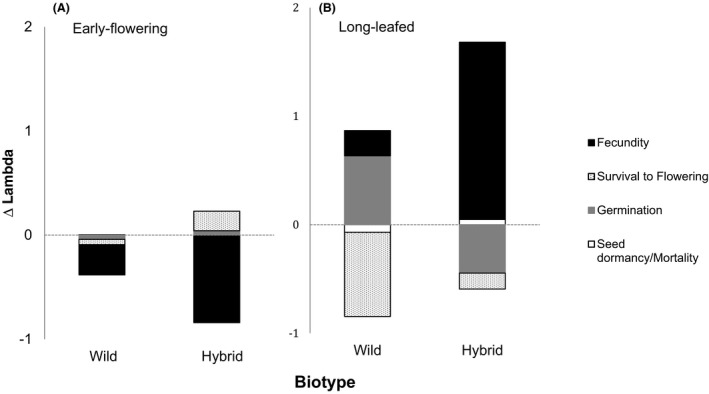
Contributions from seed dormancy/mortality, germination, flowering and fecundity vital rates to differences in lambda between control and F_5_ lineages selected for (A) early flowering or (B) long leaves in two biotypes (hybrid and wild).

Population growth rates are a consequence of contributions from each vital rate, and evaluating these consequences allows us to understand the influence of each vital rate on the relative weediness of populations. Contributions of fecundity and flowering to differences in lambda between early flowering and control populations were greater in hybrid than wild populations, whereas contributions from seeds (either remaining in the seed bank or germinating) were roughly equivalent and minor between biotypes (Fig. 2A). Fecundity negatively contributed to change in population growth rate between early flowering and control lineages for both wild and hybrid biotypes, although flowering only negatively contributed to changes in population growth rate in wild biotype populations. Contributions of fecundity to differences in lambda between large‐leafed and control populations were greater in hybrid than wild populations, whereas contributions of survival and germination were greater in wild than hybrid populations (Fig. 2B). In other words, in populations selected for early flowering, fecundity reduced population growth relative to controls (the mean matrix), but in populations selected for large leaves, fecundity increased population growth relative to control populations. Therefore, the demography of these populations differed substantially. The results from the long leaf length treatments reveal that in hybrid populations, relative to the wild populations, fecundity increased population growth, whereas germination rates reduced population growth in hybrid relative to wild lineages. Fecundity positively contributed to differences in lambda between large‐leafed and control lineages for both wild and hybrid biotypes, whereas survival to flowering only negatively contributed to differences in lambda in wild biotype populations and the direction of contributions from germination to differences in lambda were affected by biotype. Across all comparisons, seed dormancy/mortality had little impact on lambda (Fig. 2).

## Discussion

Over the last two decades, questions have arisen over the evolutionary and ecological consequences of gene flow from transgenic crops to weeds, and thus weed ecologists have been asked to evaluate ongoing effects of gene flow from nontransgenic cultivars to weeds. These questions are increasingly important as crop breeding becomes more sophisticated and weedy biotypes disperse around the globe. It is likely that fitness‐enhancing traits such as resistance to pests, pathogens, and herbicides will be engineered or bred into crops more widely in the future. This research fills a significant gap in our understanding of weeds that can hybridize with crops. For instance, strong directional selection for either early flowering or long leaves did not result in crop–wild hybrid progeny that surpassed wild progeny in their expression of weediness. Further, we have, for the first time, estimated the long‐term fitness consequences of variation in life history in weeds. Identifying life‐history traits that affect population growth contributes to our understanding of which portions of the crop genome are most likely to introgress into wild populations.

Both gene flow and adaptation may contribute to positive population growth of nascent, weedy populations. From an agronomic standpoint, annual weeds that exhibit high population growth rates and also have heritable variation in key life‐history traits are likely to be most difficult to manage. Here, after three generations of random mating or artificial selection for early flowering or long leaves, we found crop–wild hybrid *Raphanus* populations tended to exhibit lower population growth rates, than nonhybrid weed populations in similar selection environments (Fig. 1D, Table 2). These differences may be due to the strength and direction of selection. Previously, we found significantly higher population growth rates in crop–wild hybrid *Raphanus* populations after three generations of natural selection (Campbell et al. 2014). Although strong, directional selection for early flowering or long leaves did not result in changes in lambda, these populations displayed significantly different demographic strategies. As a result, these highly weedy populations (i.e., early, control and large, wild and hybrid populations exhibited *λ* ≫ 1) were equally successful using different germination or survival strategies.

Weed responses to strong anthropogenic selection pressure (i.e., potentially artificial but also unintentional, human selection), including herbicide resistance or altered germination schedules due to agricultural tilling schedules, account for a large proportion of documented cases of contemporary evolution in plants (Bone and Farres 2001; Delye et al. 2013; Heap 2015). Further, a number of examples of contemporary evolution provide support for surprising amounts of long‐term crop allele introgression into weedy populations (e.g., Whitton et al. 1997; Hegde et al. 2006; Snow et al. 2010; Ellstrand et al. 2014). In contrast, some cases reveal surprisingly little introgression in wild relative populations planted near crops (Bartsch et al. 1999). Thus, empirical evidence for a strongly homogenizing role of gene flow from crops in the contemporary evolution of related wild or weedy populations is relatively common but not ubiquitous. This may be a consequence of the relative fitness of crop‐like versus weed‐like phenotypes within a weedy background. The evolution of increased success in weedy crop relatives after introduction provides convincing support that gene flow can introduce an adaptive, novel traits and thus increase population growth (Holt et al. 2004; Novack and Mack 2005). For instance, altered germination and survival of crop–wild hybrids were associated with higher relative fitness of hybrid radish in Texas, a newly invaded location (Hovick et al. 2012). Similarly, crop–wild hybridization in *Helianthus* has contributed to adaptive evolution in water stressed environments, by not only selecting for new leaf traits but also larger inflorescence size, a trait likely to change the demography of weedy sunflower population (Owart et al. 2014). Thus, rates of adaptive evolution that result in crop allele introgression depend on the rate of gene flow, the mode of inheritance of traits, and the relative fitness of heritable crop versus wild phenotypes in a weed population (Nuismer et al. 2012).

To assess the implications of weed evolution due to gene flow or selection, an LTRE perspective is useful as an LTRE approach can link change in population growth to changes in the vital rates (Fréville and Silvertown 2005). Fecundity contributed more to changes in population growth in hybrid than wild lineages. As well, large‐leafed populations tended to be more fecund than control lineages and demonstrated higher population growth. In comparison, with relatively low seed production, early flowering lineages exhibited relatively low lambdas. As rates of fecundity impose a large influence on population growth rates (Appendix 2) and as hybrid populations possess greater genetic diversity than wild populations for large size (Campbell et al. 2009a,b), the above results suggest that hybrid biotypes could evolve higher population growth rates than wild biotypes, with additional selection for large size, that would improve their relative success colonizing new environments that are subject to an array of selection pressures.

Most risk assessments of crop–wild gene flow, including our own, consider fecundity of early‐generation hybrid offspring as sufficient to assess the likelihood of persistent gene flow. Given that fecundity represented the demographic factor that contributed the most and the smallest contributions to ∆*λ*s came from seed dormancy/mortality rates, these results suggest that this may be an adequate assessment approach, rather than a full‐scale evaluation of the relative success of each demographic stage. However, germination success can seriously alter the relative fitness of genotypes (Hovick et al. 2012) and the contribution of each demographic stage to lambda can vary across years (Teitel et al. in press). Similar to our results, a LTRE conducted across 17 species of invasive and noninvasive plants revealed that invasive plant's large *λ*s were mostly attributable to sexual reproduction (Burns et al. 2013), typical of successful invasion life‐history strategy, where high fecundity allocation and plasticity is correlated with invasiveness (Daehler 2003; Morris and Doak 2004; Davidson et al. 2011). Relatively small contributions from seed dormancy suggest that attempts at suppressing seed banks may produce a less significant effect on *λ*s. This is also reflected in the relatively low elasticities observed for seed dormancies (Appendix 2). However, *Raphanus* seed banks can be dynamic and remain dormant for several years (Teitel et al. in press). A longer‐term assessment of seed bank viability as well as a more in‐depth understanding of seed below‐ground survival are needed to understand the full potential of seed dynamics for contributing to *λ*.

## Data archiving statement

The data sets used in this work have been included in Appendix 1.

## Appendix 1

Stage transitions and mortality over a growing season (2006) for artificially selected populations of *Raphanus raphanistrum* and advanced generation hybrids of *R. raphanistrum* × *R. sativus* grown in a common garden at the University of Michigan Biological Station, Pellston, MI, USA. Populations were selected for either early‐flowering (Early) or long‐leafed (Large) and compared to randomly mated control populations. Each selection treatment was replicated three times. Each value represents the number (proportion) of individuals that survived to a given demographic stage by October 2006 classified as dormant (did not emerge), died before flowering (germinated but did not flower), survived to flower (reproductive), and average number of seeds per plant. Populations were seeded at a density of 30 seeds in three replicate populations of each biotype by selection treatment by selection replicate combination.

## Appendix 2

Estimates of elasticity in vital rates measured during a growing season (2006) in artificially selected populations of *Raphanus raphanistrum* and advanced generation hybrids of *R. raphanistrum* × *R. sativus* grown in a common garden at the University of Michigan Biological Station, Pellston, MI, USA.

### Analytical methods

Elasticities, which describe how λ proportionally changes with changes to vital rates, were calculated for every individual vital rate and treatment combination in MATLAB by dividing each matrix by its dominant eigenvalue and weighting it by its sensitivity. Population growth rates and elasticities were calculated for individual replicates before averaging them across treatment combinations.

Elasticity analyses can be used to determine which life‐history transitions have the greatest effect on population growth rates. This information has been used to develop weed management strategies for key ‘choke points’ that contribute most to population growth (e.g., (Jordan et al. 1995; Parker 2000; Mertens et al. 2002; Hyatt and Araki 2006). Here, we use elasticity analyses to determine how changes in recruitment, survival, and fecundity could affect the relative invasiveness of early versus large genotypes (de Kroon et al. 2000; Caswell 2007).

### Results

Based on elasticity measures, population growth was equally responsive to small changes in germination, survival to flowering, and fecundity for wild and hybrid genotypes under control, large leaf, and early flowering selection treatments and less responsive to small changes in seed bank dynamics (see Table A2 below). Whereas population growth was equally responsive to small changes in hybrid seed bank dynamics across selection treatments, population growth of early flowering wild plants was more responsive to changes in seed bank dynamics than control, which was more, in turn, more responsive than large‐leaf selected treatments.

**Table A2:** Elasticity (e, ±SE) of *Raphanus raphanistrum* (wild) and *R. raphanistrum* × *sativus* (hybrid) population growth rate (λ) to lower level demographic parameters after evolution in response to one of three artificial selection treatments

Biotype and selection treatment	Elasticities of λ to demographic parameters			
	Dormancy	Germination	Flowering	Fecundity
Wild				
Control	0.036 ± 0.006	0.32 ± 0.002	0.32 ± 0.002	0.32 ± 0.002
Early	0.041 ± 0.01	0.32 ± 0.004	0.32 ± 0.004	0.32 ± 0.004
Large	0.020 ± 0.002	0.33 ± 0.0005	0.33 ± 0.0005	0.33 ± 0.0005
Hybrid				
Control	0.033 ± 0.003	0.32 ± 0.001	0.32 ± 0.001	0.32 ± 0.001
Early	0.037 ± 0.003	0.32 ± 0.001	0.32 ± 0.001	0.32 ± 0.001
Large	0.039 ± 0.004	0.32 ± 0.002	0.32 ± 0.002	0.32 ± 0.002
